# Berberine Suppresses Cyclin D1 Expression through Proteasomal Degradation in Human Hepatoma Cells

**DOI:** 10.3390/ijms17111899

**Published:** 2016-11-15

**Authors:** Ning Wang, Xuanbin Wang, Hor-Yue Tan, Sha Li, Chi Man Tsang, Sai-Wah Tsao, Yibin Feng

**Affiliations:** 1 School of Chinese Medicine, The University of Hong Kong, 10 Sassoon Road, Pokfulam, Hong Kong, China; ckwang@hku.hk (N.W.); hoeytan@hku.hk (H.-Y.T.); u3003781@connect.hku.hk (S.L.); 2 Laboratory of Chinese Herbal Pharmacology, Oncology Center, Renmin Hospital, Hubei University of Medicine, Shiyan 442000, China; wangxb@hbmu.edu.cn; 3 Hubei Key Laboratory of Wudang Local Chinese Medicine Research and School of Pharmacy, Hubei University of Medicine, Shiyan 442000, China; 4 Department of Anatomy, The University of Hong Kong, 21 Sassoon Road, Pokfulam, Hong Kong, China; annatsan@hku.hk (C.M.T.); gswtsao@hku.hk (S.-W.T.)

**Keywords:** berberine, Cyclin D1, ubiquitinated-dependent proteolysis, β-TrCP, tumor growth inhibition

## Abstract

The aim of this study is to explore the underlying mechanism on berberine-induced Cyclin D1 degradation in human hepatic carcinoma. We observed that berberine could suppress both in vitro and in vivo expression of Cyclin D1 in hepatoma cells. Berberine exhibits dose- and time-dependent inhibition on Cyclin D1 expression in human hepatoma cell HepG2. Berberine increases the phosphorylation of Cyclin D1 at Thr286 site and potentiates Cyclin D1 nuclear export to cytoplasm for proteasomal degradation. In addition, berberine recruits the Skp, Cullin, F-box containing complex-β-Transducin Repeat Containing Protein (SCF^β-TrCP^) complex to facilitate Cyclin D1 ubiquitin-proteasome dependent proteolysis. Knockdown of β-TrCP blocks Cyclin D1 turnover induced by berberine; blocking the protein degradation induced by berberine in HepG2 cells increases tumor cell resistance to berberine. Our results shed light on berberine′s potential as an anti-tumor agent for clinical cancer therapy.

## 1. Introduction

Overexpression of Cyclin D1 in various human cancers is regarded as a key mechanism underlying tumor angiogenesis, progression, and metastasis [1,2,3,4,5,6]. Cyclin D1 overexpression is also found to enhance cancer cells′ resistance to chemotherapeutic agents [7]. Disruption of Cyclin D1 proteolysis is one of the major mechanisms that cancer cells accumulate Cyclin D1 [8]. In particular, it was noticed that Cyclin D1 was overexpressed in hepatocellular carcinoma (HCC) and was associated with aggressive forms of HCC [9,10]. Chronic overexpression of Cyclin D1 in transgenic mice with HCC was also observed [11].

Berberine is a natural product belonging to the group of isoquinoline alkaloids that are present in many medical plants. The anti-tumor action of berberine was extensively reported, in which berberine was shown to modulate several different signal transductions to induce tumor cell cycle redistribution and apoptosis, and to inhibit tumor cell migration [12,13]. Several studies revealed that inhibitory effect of berberine on Cyclin D1 expression in various cancer cell lines including neuroblastoma SK-N-SH & SK-N-MC cells [14], human epidermoid carcinoma A431 cells [15], human prostate carcinoma LNCap, DU145 & PC-3 cells [16], human leukemia cells HL-60 [17], and pulmonary giant cell carcinoma PG cells [11], indicating that Cyclin D1 may be a potential target for berberine in cancer therapy. However, the exact mechanism of Cyclin D1 inhibition in berberine-treated cancer cells has not been well documented. A recent study reveals that berberine suppresses the activity of the AP-1 signaling pathway and decreases the binding of transcription factors to the Cyclin D1 AP-1 motif, indicating that transcriptional inhibition of Cyclin D1 may be involved in berberine′s anti-tumor effect [18]. It is interesting to examine whether the inhibitory action of berberine on Cyclin D1 expression in liver cancer cells shares the same mechanism and to figure out the exact machinery that undergoes Cyclin D1 suppression in human hepatoma cells exposed to berberine.

In this study, the underlying mechanism of Cyclin D1 suppression by berberine in human hepatoma cells was examined. It was observed that berberine could suppress both in vitro and in vivo expression of Cyclin D1 in hepatoma cells. Dose- and time-dependent Cyclin D1 inhibition is observed in HepG2 cells exposed to berberine; and the rapid ablation of Cyclin D1 induced by 6 h berberine treatment is found independent of transcriptional inhibition. We found Cyclin D1 undergoes ubiquitinated degradation in berberine-treated HepG2 cells, and phosphorylation at Thr-286 site of Cyclin D1 is required for berberine-driven Cyclin D1 degradation. The β-transducin repeat-containing protein (β-TrCP) recruitment as E3 ligases by berberine are induced when Cyclin D1 proteolyzes. Genetic depletion of β-TrCP attenuates berberine′s inductive action on Cyclin D1 degradation as well as berberine′s anti-tumor effect. Our results indicate that involvement of β-TrCP as E3 ligase in Cyclin D1 ubiquitination-dependent proteolysis is the mechanism in berberine′s inhibitory action on Cyclin D1 expression in HepG2 cells, and contributes partially to the anti-tumor action of berberine. This sheds light on berberine′s potential in the agent list for liver cancer therapy.

## 2. Results

### 2.1. Berberine Suppresses In Vitro and In Vivo Cyclin D1 Expression in Hepatoma Cells

**Figure 1:**
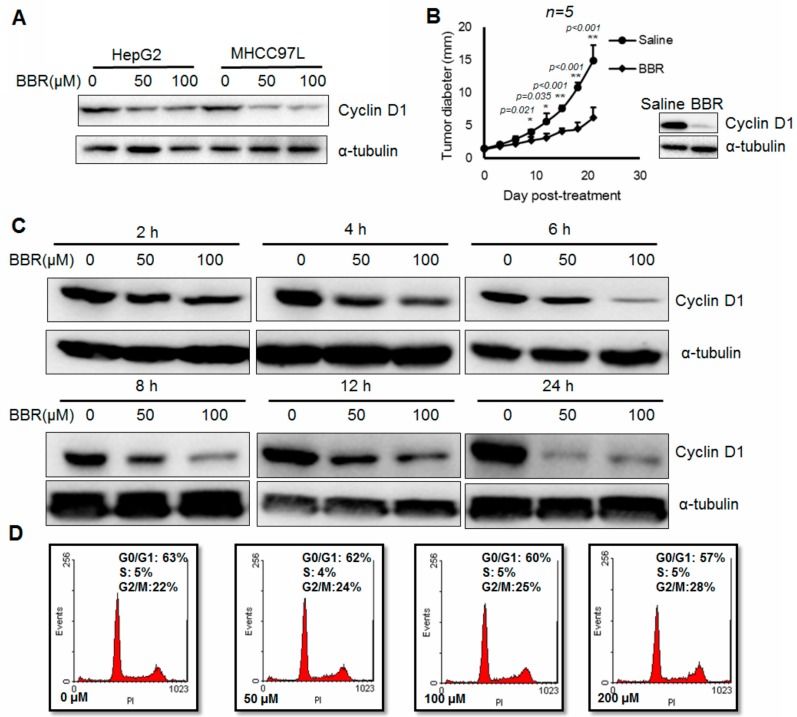
Berberine suppresses Cyclin D1 expression in hepatoma cells. (**A**) HepG2 and MHCC97L cells were treated with 100 µM berberine for 24 h. the expression of Cyclin D1 was inhibited; (**B**) Xenograft model was established as described and treatment of berberine can lead to reduced tumor size as well as Cyclin D1 expression; (**C**) Upon 6 h exposure of 100 µM berberine, the expression of Cyclin D1 was potently repressed. Cyclin D1 was detected by immunoblotting with β-actin as internal control; (**D**) HepG2 cells were treated with berberine at different doses for 6 h and then subject to cell cycle analysis. No significant cell cycle phase redistribution was observed. * *p* < 0.05,** *p* < 0.01.

It was extensively reported by our previous studies that berberine could suppress both in vitro and in vivo growth of HCC [19,20,21]. Consistently, we observed reduced expression of Cyclin D1 in hepatoma cells with berberine treatment (Figure 1A). While berberine significantly reduced proliferation of xenografted hepatoma, the expression of Cyclin D1 in hepatoma xenograft was in parallel inhibited (Figure 1B). These observations confirmed the property of berberine in suppressing in vitro and in vivo expression of Cyclin D1 in hepatoma. To further profile the action of berberine, we systemically examined Cyclin D1 expression in berberine-treated HepG2 cells. HepG2 cells with 6 h exposure to berberine exhibit significant dose-dependent reduction of Cyclin D1 expression (Figure 1C). Time-dependent manner of Cyclin D1 expression inhibition was also observed in HepG2 cells exposed to100 µM berberine (Figure 1C). Six hour exposure of 100 µM berberine to HepG2 cells was unable to carry out any alteration on the cell phase distribution, indicating that the rapid suppression on Cyclin D1 is not attributed to the cycle arrest induction by berberine in HepG2 cells (Figure 1D). This observation indicates that Cyclin D1 inhibition may occur prior to cell cycle change and can cause redistribution of cell cycle phases. Our findings reveal that berberine could rapidly inhibit Cyclin D1 expression in time- and dose-dependent manner but is independent on cell cycle.

### 2.2. Berberine Triggers Post-Translational Suppression on Cyclin D1 Expression

**Figure 2:**
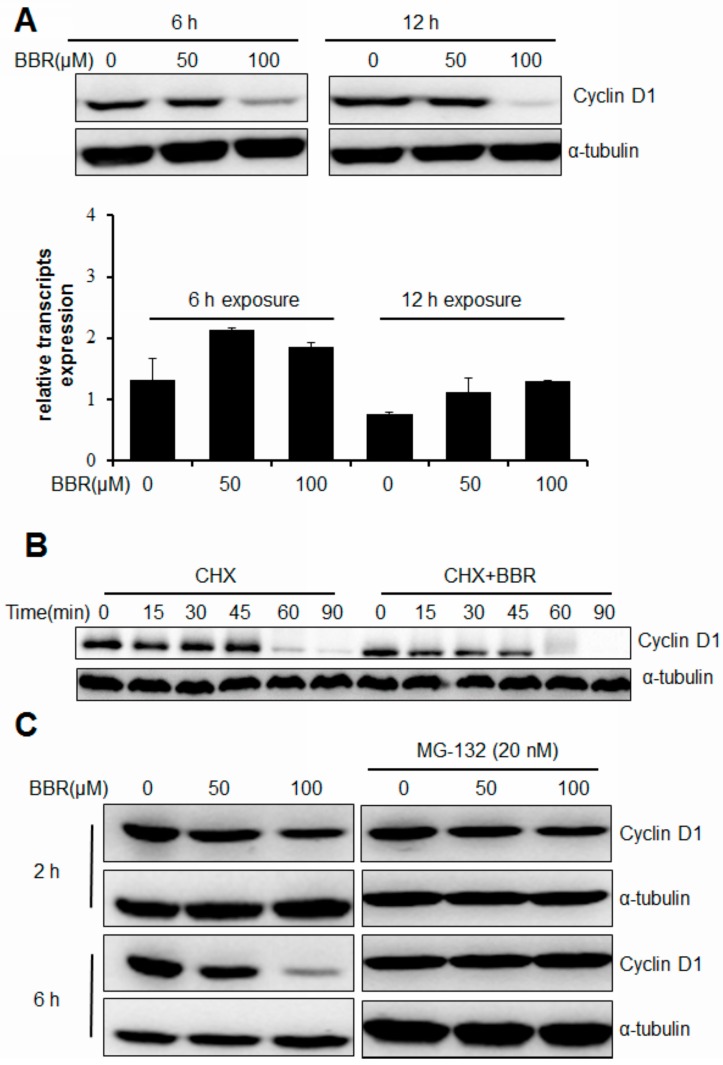
Berberine inhibits Cyclin D1 expression in HepG2 cells via post-translational control. (**A**) qPCR was used to detected the mRNA transcript of Cyclin D1 with GAPDH as internal control. No mRNA changed while Cyclin D1 protein was reduced by berberine; (**B**) Cells were treated with berberine in the presence of 150 µg/ml Cycloheximide. Reduced half-life in berberine-treated cells were found; (**C**) Cells were treated with berberine in the presence of 20 nM MG-132. Cyclin D1 was detected by immunoblotting with α-tubulin as internal control.

A previous study reveals that berberine suppresses the activity of the AP-1 signaling pathway and decreases the binding of transcription factors to the Cyclin D1 AP-1 motif, indicating that transcriptional inhibition of Cyclin D1 may be involved in the anti-tumor effect of berberine [11]. To determine if inhibition of berberine on Cyclin D1 expression in hepatoma cells undergoes the same mechanism, we issued a quantitative real-time polymerase chain reaction (qPCR) analysis to quantify the Cyclin D1 mRNA transcripts in HepG2 cells exposed to berberine. Interestingly, we found that either 6 or 12 h exposure to berberine could not suppress the transcripts level of Cyclin D1, however, the protein expression was significantly inhibited (Figure 2A). To further examine if Cyclin D1 suppression by berberine in HepG2 cells undergoes at a post-transcriptional level, we analyzed the protein expression in HepG2 cells with or without 100 µM berberine intervention in the presence of cycloheximide, a translation and protein synthesis inhibitor. We found that 100 µM berberine could shorten the half-life of Cyclin D1 protein in the presence of 150 µg/mL cycloheximide (Figure 2B). This action is further confirmed by the observation that presence of 20 nM MG-132, a proteasome inhibitor, is able to completely block the Cyclin D1 ablation induced by berberine exposure in HepG2 cells (Figure 2C). Our results show that berberine could induce a rapid post-translational degradation of Cyclin D1 in HepG2 cells.

### 2.3. Berberine Promotes Cyclin D1 Ubiquitination in HepG2 Cells and Facilitates β-TrCP Binding

**Figure 3:**
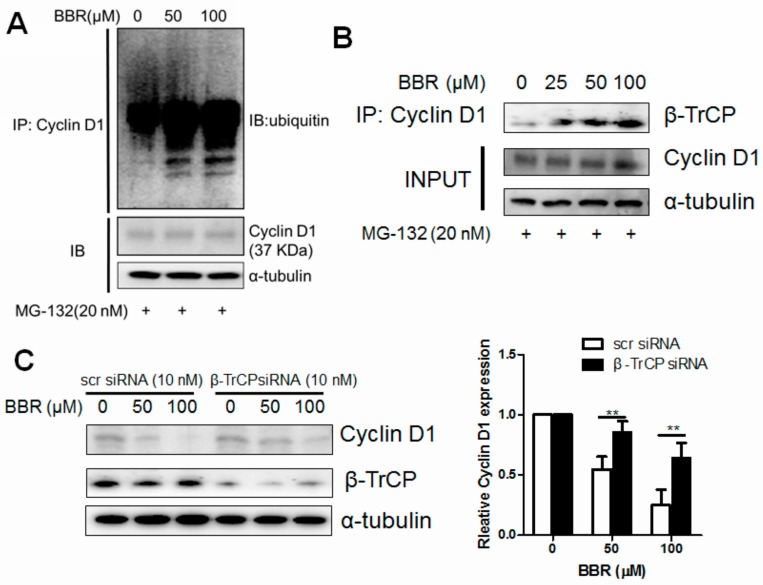
Berberine induces Cyclin D1 ubiquitination and recruits β-TrCPas an E3 ligase. (**A**) Cells were treated by berberine for 6 h in the presence of MG-132 (20 nM). Ubiquitinated Cyclin D1 was precipitated with antibody against Cyclin D1 and detected with ubiquitin antibody; (**B**) Cells were treated with berberine for 6 h in the presence of MG-132 (20 nM). Ubiquitinated Cyclin D1 was precipitated with antibody against Cyclin D1 and β-TrCP was detected with β-TrCP antibody; (**C**) shows that genetic knockdown of β-TrCP attenuates berberine′s effect on Cyclin D1 degradation. (+ means presence of the chemicals), ** *p* < 0.01.

A direct evidence of berberine-induced Cyclin D1 unbiquitination in HepG2 cells was observed (Figure 3A). The endogenous expressing Cyclin D1 in HepG2 cells with berberine treatment in the presence of 20 nM MG-132 was immunoprecipitated using specific antibody against Cyclin D1 and analyzed using antibody against ubiquitin. Increased ubiquitinated Cyclin D1 was found in a dose-dependent manner, indicating that berberine could promote the ubiquitination of endogenous Cyclin D1. We observed that one of the F-box proteins, β-TrCP, could be triggered to bind to the skp1-cullin-F-box (SCF) protein complex of Cyclin D1 upon berberine exposure (Figure 3B). As recently reported, β-TrCP could serve as an E3 ligase and be incorporated in the SCF complex-facilitating ubiquitination dependent Cyclin D1 proteolysis [22]. To figure out the direct evidence of the involvement of β-TrCP in berberine-induced Cyclin D1 ablation, we used specific siRNA against human *BTRC* gene to block its expression in HepG2 cells. Partial genetic deletion of β-TrCP in HepG2 cells attenuates berberine′s action on Cyclin D1 expression (Figure 3C). Our results may indicate that β-TrCP serves as the particular E3 ligase in berberine-driving Cyclin D1 proteolysis in HepG2 cells.

### 2.4. Berberine Promotes Cyclin D1 Phosphorylation and Nuclear Export in HepG2 Cells

**Figure 4:**
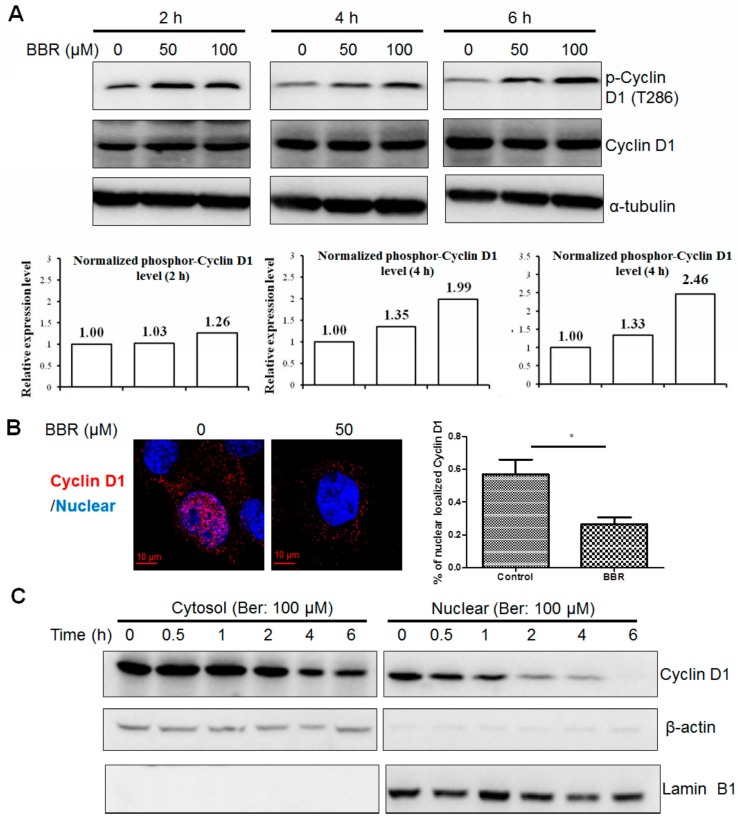
Berberine induces Cyclin D1 phosphorylation at T286 site and its nuclear export in HepG2 cells. (**A**) Cells were treated with berberine in the presence of MG132. The expression of phosphor-Cyclin D1 was normalized by total Cyclin D1 to avoid fluctuation induced by dynamic degradation of Cyclin D1; (**B**) Cells were treated with berberine for 6 h and fixed. Cyclin D1 was stained (Red) and DAPI was used to stain the nucleus; (**C**) Cells were treated with berberine and cytosolic and nuclear fractions were collected. β-actin and Lamin B1 were used as internal controls, respectively. * *p* < 0.05.

Previous studies reported that Cyclin D1 turnover was mediated by ubiquitin-dependent proteasomal degradation and dependent on T286 (the threonine 286) phosphorylation [23]. However, it was observed that certain mutations stabilized Cyclin D1 but did not affect its polyubiquitylation, which could prove that the regulation of Cyclin D1 degradation may be ubiquitin-independent [24]. Identifying if the berberine-induced Cyclin D1 degradation in HepG2 is dependent on the phosphorylation on its T286 site, we first examined if berberine could promote the Cyclin D1 phosphorylation in HepG2 cells. Western blot analysis indicates that berberine-facilitated Cyclin D1 repression in HepG2 cells was accompanied with increases in Thr-286 phophorylation in the presence of MG132, the proteasome inhibitor (Figure 4A), and the effect of berberine in triggering Cyclin D1 phosphorylation in HepG2 cells is in dose- and time-dependent manner. This indicates that phosphorylation of Cyclin D1 at the T286 site may be involved in its degradation induced by berberine. Since the ubiquitination process of Cyclin D1 is conducted in cytoplasm, the nuclear export is necessary for berberine-facilitated Cyclin D1 degradation. Both immunofluorescence and immunoblotting analysis exhibit that berberine could reduce the nuclear localization of Cyclin D1 in HepG2 cells (Figure 4B,C). These data suggest that the ability of berberine to promote phosphorylation dependent nuclear transport and ubiquitination of Cyclin D1 plays an integral role in its subsequent degradation.

### 2.5. Berberine-Induced Cyclin D1 Degradation Is T286 Phosphorylation Dependent

**Figure 5:**
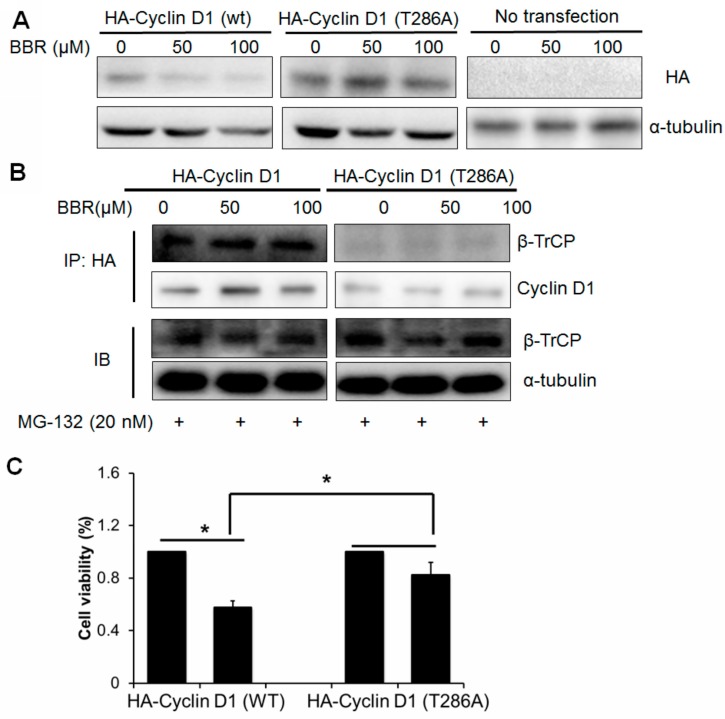
T286 phosphorylation is required from Cyclin D1 ubiquitin-proteasomal degradation induced by berberine. (**A**) Cells expressing HA-tagged wt and mutant Cyclin D1 was treated with berberine for 6 h. Expression of exogenous Cyclin D1 was blotted with hemagglutinin (HA) antibody; (**B**) Cells expressing HA-tagged wt and mutant Cyclin D1 was treated with berberine for 6 h. The protein complex was precipitated with HA antibody and precipitated β-TrCP and Cyclin D1 were detected. INPUT level of β-TrCP was detected as control; (**C**) Cells expressing HA-tagged wt and mutant Cyclin D1 was treated with berberine for 24 h. Cell viability was detected by 3-(4,5-dimethylthiazol-2-yl)-2,5-diphenyltetrazolium bromide (MTT) assay (+ means presence of the chemicals). * *p* < 0.05.

In order to determine if phosphorylation of Cyclin D1 at T286 site is required for its degradation induced by berberine in HepG2 cells, we transfected pcDNA plasmid encoding either HA-tagged Cyclin D1 (wild-type, wt) or HA-tagged Cyclin D1 T286A mutant (mut) into HepG2 cells which were then exposed to berberine for 6 h. Immunoblotting analysis shows that the wild-type exogenous Cyclin D1 undergoes rapid degradation in the presence of berberine while mutant Cyclin D1 remains intact (Figure 5A). Since previous study reports that β-TrCP recruitment requires T286 phosphorylation of Cyclin D1, we issued that the recruitment of protein complex including β-TrCP to Cyclin D1 should be observed in cells transfected with wt Cyclin D1 but rather mut Cyclin D1. The protein complex in HepG2 cells transfected with pcDNA3 plasmid encoding either wt HA-Cyclin D1 or mut HA-Cyclin D1 T286A was precipitated by HA antibody and β-TrCP was detected by immunoblotting. Recruitment of β-TrCP was observed in HepG2 cells transfected with wt Cyclin D1 plasmid but not in cells with mut Cyclin D1 transfection when exposing to berberine, suggesting that T286 phosphorylation is required for the recruitment of β-TrCP as E3 ligase for the ubiquitination of Cyclin D1 driven by berberine (Figure 5B). This may indicate that berberine-induced Cyclin D1 ablation is T286-depdendent. To further identify the contribution of Cyclin D1 ablation in berberine′s anti-tumor action, respectively, the plasmid encoding either HA-Cyclin D1 wt or HA-Cyclin D1 T286A were transfected into HepG2 cells followed by berberine treatment and WST-1 assay was used to detect the cell response to berberine. We found that cells with expression of mut Cyclin D1 show more resistance to berberine′s effect than cells with wt Cyclin D1 transfection (Figure 5C). This indicates that cells that could not undergo T286 phosphorylation-mediated protein degradation when exposed to berberine are more likely to survive upon berberine treatment. These results exhibit that berberine induced Cyclin D1 degradation partially contributes to berberine′s effect.

## 3. Discussion

In HCC, Cyclin D1 was found overexpressed and associated with aggressive forms of HCC [9,16]. Therefore, targeting Cyclin D1 by small molecule agents may be a therapeutically relevant strategy for the treatment of Cyclin D1-overexpressing HCC [22]. As a natural product with a long history and being intensively focused on its anti-tumor activity, berberine was reported to suppress Cyclin D1 expression in various human cancer cell lines, however, few of studies reported the underlying mechanism on Cyclin D1 inhibition action of berberine. From a translational perspective, understanding how berberine-facilitated Cyclin D1 inhibition is an important and integral step in drug discovery. In our study, we found a rapid suppression action of berberine on Cyclin D1 expression in human hepatoma cells HepG2, and berberine promotes an ubiquitination-dependent proteolysis of Cyclin D1 in HepG2 cells. This kind of effect of berberine is dependent on Cyclin D1′s phosphorylation at the T286 site. Some previous studies show that berberine could upregulate the AMP-kinase and MAPK p42/p44 [25,26]. Phosphorylation of the related signaling by berberine may be responsible for its various biological functions, and our finding shows Cyclin D1 phosphorylation by berberine may be related to Cyclin D1 degradation in tumor cells. These findings suggest that the ubiquitin-proteasome signal pathway involves as a novel mechanism in Cyclin D1 ablation induced by berberine in HepG2 cells.

It was noticed that berberine can suppress the expression of Cyclin D1 in different hepatoma cell lines including HepG2 and MHCC97L. As well, Cyclin D1 was potently inhibited in berberine-treated hepatoma xenograft. The detailed mechanism of berberine in suppressing Cyclin D1 was elaborated in a particular cell line HepG2. The origin of HepG2 remains to be controversial though there are a plenty of studies that regarded it as a cell line of hepatocellular carcinoma. However, it was recently shown that HepG2 cells share more genetic similarity with hepatoblastoma but not hepatocellular carcinoma [27]. An increasing number of HCC cell lines has been developed and was used in the study of liver cancer, however, not all the cell lines have a correlation with the clinical features of liver tumor. Chen et al. compared the genomic data of tumor samples from clinical setting and that of commonly used HCC cell lines, and found that around half of cell lines have poor correlation in genetic features with human tumor samples. Fortunately, the four commonly used hepatoma cell lines, HepG2, Huh7, Hep3B, and PLC/PRF/5 exhibited high correlation to the tumors [28]. In our findings, the post-transcriptional mechanism of berberine-induced Cyclin D1 degradation was proven in one of clinically correlated cell line HepG2. The significance of this study may be increased with this mechanism being validated in other hepatoma cell lines.

**Figure 6:**
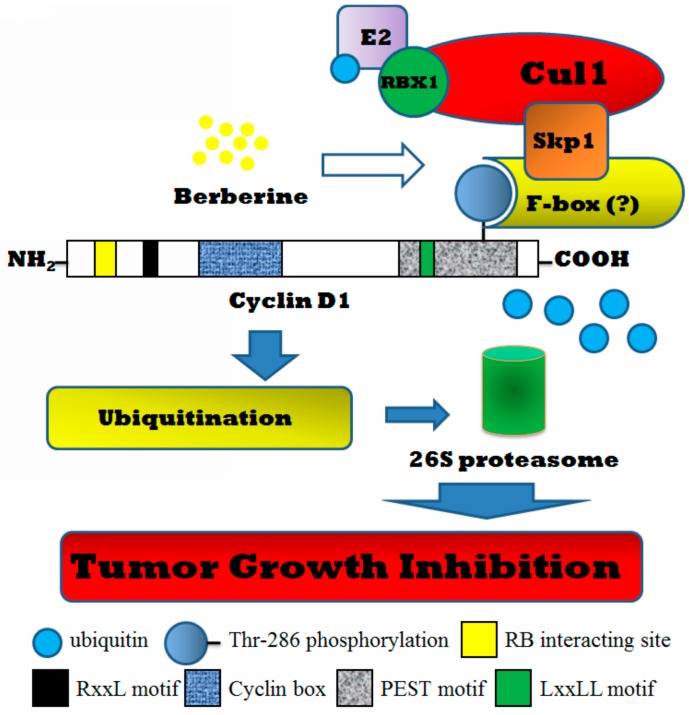
The overall scheme on the mechanism underlying berberine′s control on Cyclin D1 degradation in HepG2 cells.

The ubiquitin-proteasome dependent proteolysis is the important system in the control of protein degradation in cells [29]. The ubiquitination system is consisted of ubiquitin-activating enzymes (E1), ubiquitin-conjugating enzymes (E2), and ubiquitin-protein ligases (E3) [30,31], among which E3 is the specific enzyme for each degraded protein. For proteins controlling cell cycle, the Skp1-Cullin-F-box (SCF) complex is the particular E3 ligase for its ubiquitination [32]. β-TrCP is one of the F-box proteins that contain a protein structural motif of approximately 50 amino acids mediating protein-protein interactions. β-TrCP is linked closely to cancer for its activity in the degradation of IκBα and β-catenin [33]. Cyclin D1 was also reported as the substrate of β-TrCP in tumor cells under glucose starvation or particular anti-tumor agent treatment. Increased interaction between β-TrCP and Cyclin D1 was shown to promote Cyclin D1 protelysis in LNCap cells with exposure of peroxisome proliferator-activated receptor-γ (PPARγ) agonist STG28 and thereby contributed to its anti-tumor activity [22]. In our study, we observed that berberine, a natural product with wide spectrum of anti-tumor activity, could promote the recruitment of SCF protein complex and Cyclin D1 in HepG2 cells and facilitate Cyclin D1 proteolysis. We found that Cyclin D1 expression inhibition by berberine is dependent on ubiquitination pathway, and the particular F-box protein β-TrCP is involved. Knockdown of β-TrCP expression attenuates the Cyclin D1 turnover induced by berberine in HepG2 cells in a dose-dependent manner, indicating that β-TrCP plays a key role in berberine′s action. Moreover, genetic deletion of β-TrCP partially increases the viability of HepG2 cells with exposure of berberine, revealing that Cyclin D1 degradation induced by berberine may contribute partially to its anti-tumor activity. The overall scheme of the mechanism underlying berberine′s action on Cyclin D1 degradation is shown in Figure 6. We found that long-termed treatment of berberine increases its potency in suppressing tumor cell growth as well as in potentiating Cyclin D1 turnover. Our findings in this study indicate berberine′s potential as an anti-tumor agent with clear mechanism in inducing Cyclin D1 degradation.

## 4. Materials and Methods

### 4.1. Chemicals and Plasmids

Berberine chloride, protein synthesis inhibitor cycloheximide and proteasome inhibitor MG-132 were purchased from Sigma-aldrich (St. Louis, MO, USA). Plasmid pcDNA3 Cyclin D1-HA (Plasmid 11181) and pcDNA3 Cyclin D1-HA (T286A, Plasmid 11182) were kindly provided by Bruce Zetter (Harvard Medical School, deposited by Addgene, Cambridge, MA, USA); plasmid pcDNA3 HA-ubiquitin (Plasmid 18712) was provided by Edward Yeh (The University of Texas-Houston Health Science Center, deposited by Addgene).

### 4.2. Cell Line and Cell Culture

The human hepatoma cell line HepG2 was obtained from American Type Culture Collection (ATCC, Manassas, VA, USA). MHCC97L cells were kindly gifted by Man Kwan from Department of Surgery, The University of Hong Kong (Hong Kong, China). Cells were maintained in the high glucose Dulbecco′s Modified Eagle Medium (DMEM, Invitrogen, Carlsbad, CA, USA) supplemented with 10% FBS (Invitrogen), and incubated in a humidified atmosphere containing 5% CO_2_ at 37 °C.

### 4.3. Xenograft Model

The protocol for animal study was approved by the Committee on the Use of Live Animals in Teaching and Research (CULATR) of the University of Hong Kong (code: 2441-11). Animal was housed in Laboratory Animal Centre of The University of Hong Kong with humane care. Four-week-old female BALB/c nude mice received 1 × 10^6^ MHCC97L cells by subcutaneous injection at the right flank. One week after injection, mice were randomized into two groups. The treatment group of mice received intraperitoneal injection of berberine (10 mg/kg/2 days) while mice in control group received the same volume of saline buffer. Treatment lasted three weeks and at the end of study, mice were sacrificed by overdose of pentobarbital (200 mg/kg) and tumor was dissected out for analysis.

### 4.4. Real-Time Quantitative Polymease Chain Reaction

Total RNA was extracted and purified using RNeasy Mini Kit (Qiagen, Hilden, Germany) following the manufacturer′s instruction. Reverse-transcription reaction was performed using QuantiTech Reverse Transcription Kit (Qiagen) to prepare cDNA samples. The quantitative real-time PCR (qRT-PCR) was conducted by QuantiTect SYBR Green PCR Kit (Qiagen) with 1 µM primers for *CCND1* (right: 5′-GACCTCCTCCTCGCACTTCT-3′; left: 5′-GAAGATCGTCGCCACCTG-3′; Invitrogen, USA) on LightCycler 480 real-time PCR system (Roche, Basel, Switzerland). The expression of *GAPDH* was used as endogenous control (right: 5′-GCCCAATACGACCAAATCC-3′; left: 5′-GCTAGGGACGGCCTGAAG-3′ Invitrogen, USA) for the normalization of gene expression of *CCND1*.

### 4.5. Cell Cycle Analysis

HepG2 cells exposed to berberine (0, 50, 100 µM) for 6 h were collected and fixed in ice-cold 70% ethanol overnight. Cells were then centrifuged for 5 min at 1500 rpm at room temperature. Ethanol was discarded and cell pellet was re-suspended in PBS containing propidium iodide (5 µg/mL) and RNase A (50 units/mL). Cell cycle phase distribution were examined by flow cytometer (Epics XL, Beckman Coulter, Brea, CA, USA) and analyzed by Winmidi V2.9 program.

### 4.6. Immunofluoscence

HepG2 cells were seeded in 10 mm cover slip and incubated overnight. Then cells were treated with berberine (0, 50, 100 µM) for 6 h. Cells were fixed in 4% paraformaldehyde for 1 h and then penetrated in 0.1% Triton-X100 for 15 min. Cells were blocked in 5% normal goat serum in PBS overnight at 4 °C followed by incubation with Cyclin D1 primary antibody (1:50) overnight at 4 °C. After washing, the bound primary antibody was detected using Texas Red goat anti-rabbit antibody (Santa Cruz, 1:200) at room temperature for 2 h. The nuclear counterstaining was performed using a 4,6-diamidino-2-phenylindole-containing mounting medium (Invitrogen) before examination. Images were taken using confocal microscope (Carl Zeiss, Oberkochen, Germany, 400 magnification, CCD camera).

### 4.7. Subcellular Fractionation

Cells were lysed with cold hypotonic buffer (10 mM Hepes, 10 mM KCl, 0.1 mM EDTA, 0.4% NP-40, 0.05 mM DTT) containing protease inhibitor cocktail (Roche) for 5 min and then supernatant (Cytoplasmic fraction) was collected by centrifugation at 14,000× *g* 4 °C. The residue was then extracted with nuclear extraction buffer (20 mM Hepes, 400 mM NaCl, 1 mM EDTA, 0.05 mM DTT, in the presence of protease inhibitor cocktail) on ice for 30 min, followed by centrifugation at 14,000× *g* for 10 min at 4 °C. Supernannt was collected as nuclear fraction. Both cytoplasmic and nuclear fraction was separated and immunoblotted with β-actin and LAMIN B1 as control, respectively [22].

### 4.8. Immunoblotting

Protein was isolated on SDS-PAGE and then transferred to polyvinylidene fluoride membrane (PVDF, Biorad, Hercules, CA, USA). The membrane was then blocked with 5% BSA overnight at 4 °C, followed by incubation with respective primary antibodies overnight at 4 °C. After washing, the membrane was then incubated with appropriate secondary antibody (Abcam, Cambridge, UK) at room temperature for 2 h. Image was captured using a chemiluminenescence imaging system (Bio-rad, Biorad) with ECL advanced kit (GE Healthcare, Little Chalfont, UK) as substrate.

### 4.9. Co-Immunoprecipitation Assay

Cells were treated with berberine in the presence of MG-132 for 6 h. Collected cell pellets were extracted using NP-40 lysis buffer (Invitrogen) supplemented with cocktail protease inhibitor (Roche) for 5 min on ice followed by centrifuging at 14,000 rpm at 4 °C for 10 min. The supernatant was collected and aliquoted. Co-immunoprecipitation assay was performed using Dynabeads^®^ protein G kit (Invitrogen) following manufacturer′s instruction. Briefly, each 1.5 mg of magnetic beads were transferred to a 1.5 mL microcentrifuge tube and separated on the magnet (Millipore, Billerica, MA, USA) to remove the supernatant. Diluted antibodies were bound by incubating with the beads for 10 min at room temperature with rotation. The beads were collected by placing the tube on the magnet and removing the supernatant. The cell lysate was then incubated with antibody-bound beads for 10 min at room temperature with rotation and then discarded. After washing, the bound protein was eluted by incubating the beads with 20 µL elution buffer for 2 min at room temperature with rotation. The supernatant was collected and the eluted proteins were denatured and analyzed by immunoblotting.

### 4.10. RNA Interference

HepG2 cells were seeded in DMEM medium supplemented with 10% FBS and 1% antibiotics with 70% confluence. 24 h before transfection, medium was discarded and replaced with serum- and antibiotic-free DMEM medium. Transfection was carried out using Lipofectamine 2000 (Invitrogen) according to manufacturer′s instruction. 10 µg of siRNA against human β-TrCP (sc-37178, Santa Cruz, CA, USA) was transfected. The cells were supplemented with DMEM medium with 10% FBS and 1% antibiotics 6 h after transfection. Treatment of berberine was conducted within 48 h after transfection.

### 4.11. Statistical Analysis

All experiments were conducted in triplicate except particular notice. Results were analyzed using student *t*-test and expressed as mean ± SD.

## 5. Conclusions

In conclusion, we observed that berberine exhibits dose- and time-dependent inhibition on Cyclin D1 expression in human hepatoma cells. Berberine increases the phosphorylation of Cyclin D1 at Thr286 site, and recruits the SCFβ-TrCP complex to facilitate Cyclin D1 ubiquitin-proteasome dependent proteolysis. In addition, berberine potentiates Cyclin D1 nuclear export to cytoplasm for proteasomal degradation. Knockdown of β-TrCP blocks Cyclin D1 turnover induced by berberine; blocking the protein degradation induced by berberine in HepG2 cells increases tumor cell resistance to berberine. Our results shed light on berberine’s potential as an anti-tumor agent for clinical cancer therapy.
